# Porcine Hemagglutinating Encephalomyelitis Virus and Respiratory Disease in Exhibition Swine, Michigan, USA, 2015

**DOI:** 10.3201/eid2307.170019

**Published:** 2017-07

**Authors:** Joshua N. Lorbach, Leyi Wang, Jacqueline M. Nolting, Madonna G. Benjamin, Mary Lea Killian, Yan Zhang, Andrew S. Bowman

**Affiliations:** The Ohio State University, Columbus, Ohio, USA (J.N. Lorbach, J.M. Nolting, A.S. Bowman);; Ohio Department of Agriculture, Reynoldsburg, Ohio, USA (L. Wang, Y. Zhang);; Michigan State University, East Lansing, Michigan, USA (M. Benjamin);; National Veterinary Services Laboratories, Ames, Iowa, USA (M.L. Killian)

**Keywords:** porcine hemagglutinating encephalomyelitis, porcine hemagglutinating encephalomyelitis virus, coronavirus, swine, influenza A virus, disease outbreaks, differential diagnosis, viruses, exhibition swine, respiratory infections, Michigan, Ohio, Indiana, United States

## Abstract

Acute outbreaks of respiratory disease in swine at agricultural fairs in Michigan, USA, in 2015 raised concern for potential human exposure to influenza A virus. Testing ruled out influenza A virus and identified porcine hemagglutinating encephalomyelitis virus as the cause of influenza-like illness in the affected swine.

The commingling of pigs and humans at agricultural fairs has been responsible for most zoonotic influenza A virus (IAV) cases over the past 5 years. During routine IAV surveillance in exhibition swine in the summer of 2015, influenza-like illness (ILI) was noted in swine at 6 of 14 agricultural fairs surveilled in Michigan, USA. Acute outbreaks of ILI in swine at 2 fairs were so severe that animal health and fair officials closed the swine barns to nonessential personnel out of concern for potential human exposure to IAV. Nasal swab specimens were collected from representative swine and tested for IAV (Technical Appendix). IAV was not detected in samples from the pigs at any of the Michigan fairs; however, next-generation sequencing (NGS) identified porcine hemagglutinating encephalomyelitis virus (PHEV) in a specimen from a clinically ill pig. 

Following the initial PHEV detection, all samples from the 14 Michigan fairs held in 2015 were screened for the coronavirus; PHEV was detected at 10 (71.4%) of the 14 fairs, with 108 (38.7%) of 279 pigs testing positive. Given the high prevalence of PHEV in Michigan exhibition swine and the uncommon clinical presentation for PHEV (i.e., ILI in market-age pigs), we initiated further epidemiologic investigation. 

## The Study

We screened nasal swabs from pigs at 14 Ohio fairs and 14 Indiana fairs for PHEV (detailed methods in Technical Appendix). Pigs at 4 of the Indiana fairs and 5 of the Ohio fairs exhibited signs of respiratory disease. We detected PHEV in 4 (14.3%) of 28 Ohio and Indiana fairs; 23 (4.1%) of 560 pigs tested positive. The increased risk of PHEV detection in samples from Michigan exhibition swine compared with samples collected from pigs in Ohio and Indiana (risk ratio 9.4, 95% CI 6.2–14.4) indicated epizootic behavior of PHEV in the Michigan fairs.

**Figure 1:**
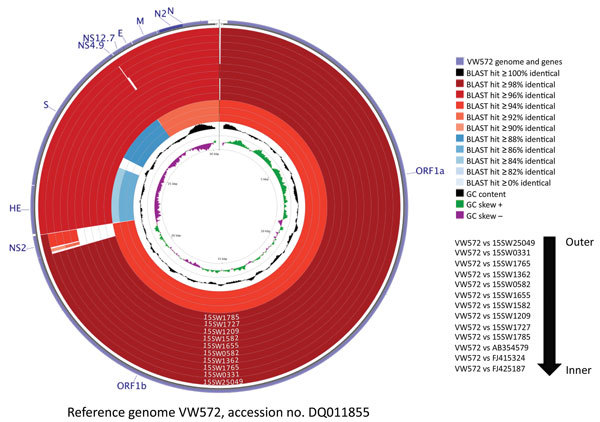
Genomic comparison of 10 porcine hemagglutinating encephalomyelitis virus (PHEV) strains from fairs in Michigan, Indiana, and Ohio, USA, 2015, to 3 non-PHEV coronavirus (CoV) strains from GenBank (bovine CoV Kakegawa, accession no. AB354579; human enteric CoV 4408, accession no. FJ415324;, white-tail deer CoV WD470, accession no. FJ425187) and a reference genome from a PHEV strain from Belgium (VW572, accession no. DQ011855). Analysis was completed by using CGView Comparison Tool software (*1*). The corresponding strain/sample for rings are detailed on the right. The innermost 2 rings display GC content and GC skew. NS2, nonstructural protein 2; ORF, open reading frame.

**Figure 2:**
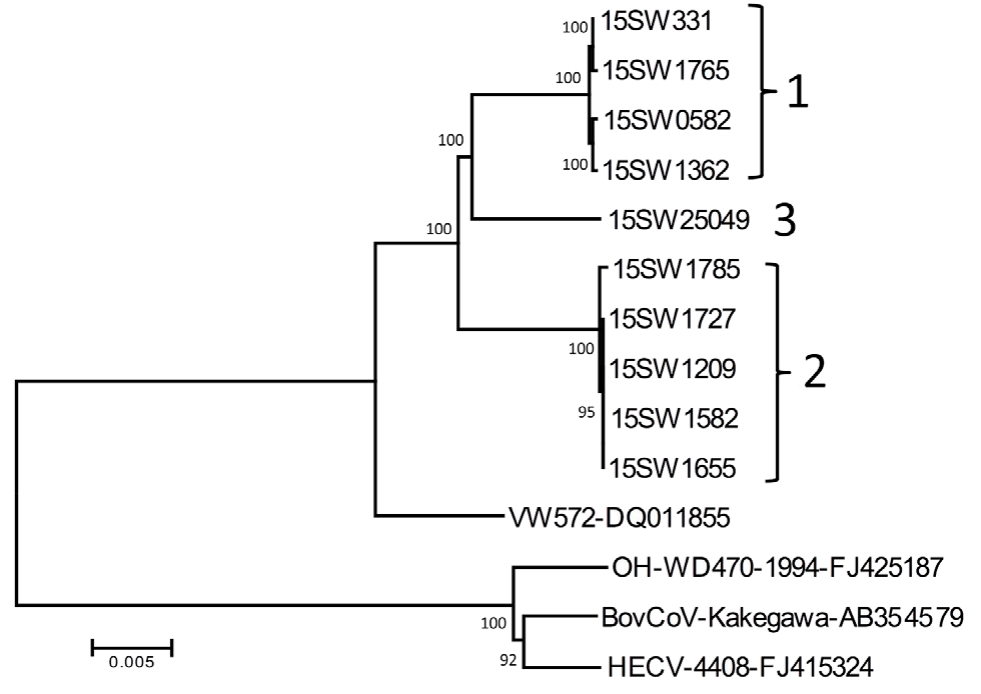
Phylogenetic tree constructed on the basis of the whole-genome sequence of porcine hemagglutinating encephalomyelitis virus (PHEV) strains from fairs in Michigan, Indiana, and Ohio, USA, 2015 (indicated by genotype labels at right), compared with bovine CoV (BovCoV), human enteric CoV (HECV), and white-tail deer CoV and a reference PHEV strain from Belgium (VW572). Reference sequences obtained from GenBank are indicated by strain name and accession number. Numbers along branches indicate bootstrap values. Scale bar indicates nucleotide substitutions per site. CoV, coronavirus.

Although PHEV has been recognized for decades, few PHEV genomes have been publicly deposited. We performed NGS on representative PHEV-positive samples to investigate genetic diversity; 10 complete sequences and 1 partial sequence were obtained. Sequence analysis showed that the 10 complete PHEV strains had 2.1%–2.2% genome difference from a PHEV strain from Belgium (VW572) and 7.2%–7.4% genome difference from human enteric coronavirus (CoV) (HECV) 4408, bovine CoV Kakegawa, and white-tail deer CoV WD470 (Figure 1). Complete genome, nonstructural 2 (NS2) gene, spike gene, and NS4.9 gene phylogenetic analyses indicated 3 distinct clusters, referred to as genotypes 1–3, based on deletions in the NS2 gene (Figure 2; Technical Appendix). It is likely that the deletions observed in this study contribute to viral evolution and may confer respiratory tropism of PHEV because deletion patterns are common in the genome of porcine respiratory CoV, which has a strong respiratory tropism. In contrast, the 3 other porcine CoVs have a strong enteric tropism (transmissible gastroenteritis virus, porcine epidemic diarrhea virus, and porcine deltacoronavirus). 

The presence of ILI in pigs at multiple Michigan fairs, along with the increased risk of PHEV detection at these fairs, supports a causal link between PHEV and respiratory disease. PHEV is a single-stranded positive-sense RNA coronavirus belonging to the family *Coronaviridae*, genus *Betacoronavirus*. The virus is 1 of 5 known porcine CoVs causing disease in swine and is considered endemic worldwide, where it maintains itself by successively infecting groups of animals after replacement or weaning (*2**,**3*). PHEV typically affects pigs <3 weeks of age; clinical syndromes include vomiting and wasting disease and encephalomyelitis (*4**,**5*). Upper respiratory tract and pulmonary lesions have rarely been described (*6*); however, the primary route of PHEV infection is through upper respiratory tract epithelium. Sneezing and coughing may be the first clinical signs observed in piglets, supporting our premise that PHEV may cause respiratory disease in older swine (*2*). Although there are no data to definitively prove this premise, a previous report suggested an association between PHEV and clinical disease in older animals (*1*). A confounder at breeding facilities is the presence of animals of multiple age groups and bias toward recognizing the classical disease in piglets; our data represent a relatively homogenous group of market-age pigs.

Our findings also appear to highlight a distinct transmission network within Michigan exhibition swine; despite geographic contiguity and no barriers to interstate travel, Michigan samples, compared with those from Ohio and Indiana, yielded different proportions of PHEV detection. This finding is further supported by the observation that PHEV sequenced from Michigan fairs was predominantly genotype 2, which was not detected in Ohio or Indiana (Table). Such a transmission network may be the result of common routes of travel or sites of commingling of swine, including larger swine exhibitions before county fairs. Animal networks have been described in additional species and locations and are not unique to the Michigan fairs (*8**,**9*).

**Table:** PHEV obtained from samples of swine at fairs in Michigan, Indiana, and Ohio, USA, 2015*

Fair	Total no. samples	No. PHEV positive	Risk for PHEV positivity	ILI	Strain name	Genotype	GenBank accession no.
Michigan							
A	20	9	0.45	Yes	PHEV-CoV USA-15SW1727	2	KY419111
B	20	20	1.00	Yes	PHEV-CoV USA-15SW1362	1	KY419110
C	20	10	0.50	Yes	PHEV-CoV USA-15SW1582	2	KY419113
D	20	20	1.00	Yes	PHEV-CoV USA-15SW1655	2	KY419109
E	19	7	0.37	Yes	PHEV-CoV USA-15SW25049	3	KY419103
F	20	19	0.95	Yes	PHEV-CoV USA-15SW1209	2	KY419107
G	20	9	0.45	No	NA	NA	NA
H	20	9	0.45	No	NA	NA	NA
I	20	4	0.20	No	PHEV-CoV USA-15SW1785	2	KY419106
J	20	1	0.05	No	PHEV-CoV USA-15SW24992†	2	KY419108
All others, n = 4	80	0	0.00	No	NA	NA	NA
Total, n = 14	279	108	0.39				
Indiana							
K	20	1	0.05	No	NA	NA	NA
L	20	8	0.40	No	PHEV-CoV USA-15SW1765	1	KY419112
M	20	1	0.05	No	PHEV-CoV USA-15SW0331	1	KY419104
All others, n = 11	220	0	0.00	Yes (4)	NA	NA	NA
Ohio							
N	20	13	0.65	No	PHEV-CoV USA-15SW0582	1	KY419105
All others, n = 13	260	0	0.00	Yes (5)	NA	NA	NA
Ohio/Indiana							
Total, n = 28	560	23	0.04				

*Reverse transcription PCR PHEV detection results are shown with calculated risk. Aggregate data for Michigan or Ohio and Indiana are shown. PHEV strain name and corresponding GenBank accession numbers are listed for samples that yielded sequence data. ILI, influenza-like illness; NA, not applicable; PHEV, porcine hemagglutinating encephalomyelitis virus. †Partial genome (nonstructural protein 2).

During our interpretation of the data, we considered several limitations. First, no tissues were available to demonstrate pathologic lesions associated with the virus. However, NGS failed to detect the presence of additional pathogens aside from a single sample that contained porcine parainfluenza virus type 1 in addition to PHEV. Second, detection of PHEV in samples could not be directly correlated with respiratory disease in individual animals due to assessment of ILI at the fair level. However, at the fairs where barns were closed because of concerns of IAV, most swine were affected by ILI, and >50% of pigs were PHEV positive at these locations. Last, it is difficult to conclude whether genotype 2 PHEV was present in Ohio and Indiana, because not all positive samples were sequenced. Although the genotype data may not be statistically significant because of the small sample size, there appears to be a clear difference in genotype distributions.

## Conclusions

Our findings provide strong evidence for the role of PHEV as a respiratory pathogen and genomic characterization of clinically relevant strains circulating in US swine herds. The ILI in swine in this study is considered an atypical presentation of PHEV and may reflect unique presentation of PHEV in older but naive swine populations, an atypical form of disease, or increased virulence. Guarded interpretation of our data suggests that, at minimum, PHEV should be considered as a differential diagnosis in clinical outbreaks of ILI in market-age swine. Future surveillance and research are needed to further investigate the association of PHEV with respiratory disease in commercial and exhibition swine.

The rapid government and local authority responses to the outbreaks of respiratory disease in pigs at the fairs involved in our study was justified given the public health threat of IAV. Variant IAV (H3N2v) was responsible for many human cases, including 1 death, during outbreaks in 2011–2016 in which swine-to-human transmission was demonstrated (*10**,**11*). Most recently, in August 2016 there was a regional outbreak of H3N2v virus infection in 18 persons with recent exposure to swine at 7 fairs in Michigan and Ohio (*11*). PHEV is not known to cause any disease in humans, but the PHEV transmission network uncovered in Michigan during 2015 may represent a pathway for both intraspecies and interspecies transmission of additional pathogens, including IAV. Although it is inappropriate to make leaps in assuming that this report could predict such a future outbreak, it is worth contemplating potential outcomes had the animals been transmitting a zoonotic agent such as IAV.

Technical Appendix Discussion of methods and results of pathogen detection and genomic sequencing of porcine hemagglutinating encephalomyelitis virus in exhibition swine, Michigan, USA, 2015.
