# Supplementary material for: Porcine Hemagglutinating Encephalomyelitis Virus and Respiratory Disease in Exhibition Swine, Michigan, USA, 2015
Source: Emerg Infect Dis. 2017 Jul;23(7):1168–71. doi: 10.3201/eid2307.170019 (PMC5512476; doi:10.3201/eid2307.170019)
Supplement: Technical Appendix — Discussion of methods and results of pathogen detection and genomic sequencing of porcine hemagglutinating encephalomyelitis virus in exhibition swine, Michigan, USA, 2015. [file 17-0019-Techapp-s1.pdf]

Article DOI: https://doi.org/10.3201/eid2307.170019

Porcine Hemagglutinating Encephalomyelitis Virus and Respiratory Disease in Exhibition Swine, Michigan, USA, 2015

Technical Appendix

Methods

Study Sites and Sampling

During the summer of 2015, an ongoing influenza A virus (IAV) surveillance program targeting exhibition swine was expanded to include 14 Michigan fairs. At the conclusion of swine exhibition activities and before departure of swine from enrolled fairs, study team members visually observed the swine for clinical signs of respiratory disease and collected nasal swab samples. Twenty specimens per fair were targeted to provide 95% confidence of IAV detection, assuming a prevalence $\geq$15%. Individual nasal swab specimens were placed in vials containing brain heart infusion media supplemented with penicillin G and streptomycin and frozen at –80°C until processing. A total of 279 nasal swab specimens were collected from the 14 Michigan fairs. The Ohio State University Institutional Animal Care and Use Committee approved the use of animals in this study under protocol no. 2009A0134.

Pathogen Detection and Epidemiology

We extracted RNA from the samples using Mag-Bind Viral DNA/RNA 96 Kit (Omega, Norcross, GA, USA) and MagMAX Express-96 Deep Well Magnetic Particle Processor (ThermoFisher, Waltham, MA, USA). We initially screened the samples for IAV by real-time reverse transcription PCR (rRT-PCR) with a commercially available kit (VetMAX-Gold SIV Detection Kit; Applied Biosystems, Austin, TX, USA) using manufacturer-provided protocols. We screened samples for porcine reproductive and respiratory syndrome virus (PRRSV) by rRT-PCR using described methods (*1*).

We selected a sample taken from a Michigan exhibition pig with clinical respiratory disease typical for this outbreak and negative for IAV and PRRSV for de novo sequencing to identify potential pathogens within the specimen. We prepared an RNA library using Ion Total RNA-Seq Kit version 2 (ThermoFisher) according to manufacturer instructions. We amplified the library with the Ion PGM Hi-Q Chef Kit on the Ion Chef System and sequenced on the Ion Personal Genome Machine System. Following the detection of porcine hemagglutinating encephalomyelitis virus (PHEV), we screened all remaining samples from pigs at Michigan fairs for PHEV by rRT-PCR using specific primers and protocol provided by the University of Minnesota Veterinary Diagnostic Laboratory (protocol MOL.SOP.291).

IAV surveillance activities during 2015 involved agricultural fairs beyond Michigan, including those taking place in Ohio and Indiana. We selected samples from 14 fairs from each of those states (n = 280 per state) for PHEV screening to compare them to the prevalence in Michigan exhibition swine. Fairs from Ohio and Indiana were selected based on influenza-like illness (ILI) in the pigs, corresponding time to Michigan fairs, and IAV-negative swine samples. Using the pooled results in Ohio and Indiana (OH/IN), we compared the risk of PHEV detection both between Michigan and OH/IN individual samples and between Michigan and OH/IN fairs. We calculated risk ratio (RR) for individual samples and individual fairs, and constructed 95% CIs. We calculated p values using Fischer’s exact probability test (*2*).

Genomic Characterization and Comparison

We selected 1 sample per fair where PHEV was detected for sequencing on the basis of the lowest PHEV rRT-PCR cycle threshold value at each fair (n = 14). We applied a sequence-independent, single-primer amplification method to amplify the genome of PHEV from extracted RNA. We used the Nextera XT DNA Library Preparation Kit (Illumina, San Diego, CA, USA) to prepare a next-generation sequencing library followed by sequencing on MiSeq. We used both Ray and SPAdes softwares for de novo assemble of 9 PHEV strains. We closed small sequencing gaps by using a Sanger sequencing method, and reamplified 2 strains (15SW0331 and 15SW0582) with a relatively smaller percentage of genome coverage (54% and 80%, respectively) by using 21 pairs of PHEV-specific primers followed by sequencing on MiSeq. We aligned and compared sequences using MUSCLE (MEGA Software version 6.06; www.megasoftware.net). We confirmed all insertions and deletions by Sanger sequencing. We performed phylogenetic comparison of the sequenced PHEV strains to reference coronavirus

sequences obtained from GenBank by complete sequence, nonstructural 2 (NS2) gene, spike gene, and NS4.9 gene. We constructed dendrograms using the neighbor-joining method with the MEGA software package. We performed bootstrap resampling (1,000 replications), and bootstrap values are indicated for each node.

Results

Pathogen Detection

Our initial screening results determined that all samples were negative for IAV. All Michigan samples were negative for PRRSV, but 1 sample from each comparison state (Ohio and Indiana) tested positive for PRRSV. De novo sequencing identified PHEV in a sample from a clinically ill Michigan exhibition pig. The resulting sequence covered 100% of the genome with an average depth of coverage of 65×, including numerous regions with <10× coverage; sequence gaps were closed with specific primers.

Genomic Sequencing

We deposited sequence data for complete and partial genomes in GenBank; previously, only 1 complete genome (PHEV-VW572) had been available in the database (Technical Appendix Figure 1). Sequence mapping analysis of raw reads showed that over 50% genome coverage for PHEV was obtained for 10 (15SW0331, 15SW0582, 15SW1209, 15SW1362, 15SW1582, 15SW1655, 15SW1727, 15SW1765, 15SW1785, and 15SW25049) out of 14 samples. Among the PHEV strains identified in the current study, the genomes range from identical to 1.8% difference, demonstrating presence of distinct strains. Further analysis reveals that the PHEV strains identified in the present study contain deletions and/or insertions in different genes, including open reading frame 1b (ORF1b), NS2, spike, NS4.9, and 3′ untranslated region (UTR) (Technical Appendix Figures 2–4).

Compared with VW572, 4 (15SW0331, 15SW0582, 15SW1362, and 15SW1765) of the 10 PHEV samples contain a 3-nt deletion (GTA) in the ORF1b at positions 20089–20091, resulting in a 1-amino-acid deletion (NEP vs. NGKP). In comparison with VW572, HECV 4408, Kakegawa, and WD470, all 10 PHEV strains have different deletion patterns in the NS2 gene, resulting in 5 different lengths of the NS2 gene. Five strains (15SW1209, 15SW1582, 15SW1655, 15SW1727, and 15SW1785) have a truncated NS2 gene, with the smallest genome

size of NS2, 61 nt, encoding only a 12-aa truncated NS2 protein (Technical Appendix Figure 5). The NS2 genes of 4 strains (15SW0331, 15SW1765, 15SW0582, and15SW1362) are 745 nt, 745 nt, 749 nt, and 751 nt in length, respectively, longer than that of VW572, but encode only 5 aa because of a premature stop codon caused by a 44-nt deletion in the 5′ NS2. 15SW25049 has the largest size of NS2 gene, 826 nt, encoding a 195-aa protein, which is 1 aa longer than that of VW572 NS2 protein.

In the case of the spike gene, compared with VW572, 9 PHEV strains have a 3-nt deletion (AAT) at positions 2680–2682, resulting in a single amino acid deletion (N, Asn), whereas 15SW25049 does not have this deletion (just as in 2 Canadian PHEV strains [67N and IAF-404]). In the case of the NS4.9 gene, 3 different deletion patterns were identified in the 10 PHEV-positive samples, as compared to VW572. The deletions result in 3 different lengths of NS4.9 (15SW0582 and 15SW1362: 13aa; 15SW0331 and 15SW1765: 28aa; 15SW1209, 15SW1582, 15SW1655, 15SW1727, 15SW1785 and 15SW25049: 20aa) (Technical Appendix Figures 6 and 7). The truncated NS4.9 protein pattern of 15SW1209, 15SW1582, 15SW1655, 15SW1727, 15SW1785 and 15SW25049 has been previously reported in 2 Canadian PHEV strains (67N and IAF-404) (*3*). In the 3′ UTR, compared with VW570, all 10 PHEV strains we detected have 1 unique single nucleotide deletion at position 174, and 5 strains (15SW0331, 15SW0582, 15SW1362, 15SW1765 and 15SW25049) have an additional single nucleotide deletion at position 190. We identified the NS2 gene of PHEV in 1 sample (15SW24992) and detected porcine parainfluenza virus-1 (PPIV-1) in 1 sample (15SW0582) in addition to PHEV.

Four strains (15SW0331, 15SW0582, 15SW1362, and 15SW1765) form genotype 1, 5 strains (15SW1209, 15SW1582, 15SW1655, 15SW1727, and 15SW1785) form genotype 2, and 1 strain (15SW25049) alone or together with VW572 or IAF-404 form genotype 3. The strain 15SW25049 (genotype 3) is more closely related to genotype 1 in the phylogenetic trees of the complete genome and NS2 gene, whereas it is more closely related to genotype 2 in the spike gene and NS4.9 gene trees. All 10 strains are distantly related to VW572, except in the NS2 gene tree, where VW572 is closely related to 15SW25049.

Overall, genomic and phylogenetic characterization demonstrated that the 10 PHEV strains are novel PHEV variants. Although deletions in the NS2 and NS4.9 genes was previously

reported in VW572, all 10 US PHEV strains reported here possess novel deletion patterns in both these genes; we also observed new deletions in ORF1b, spike gene, and 3′ UTR.

References

1. Opriessnig T, McKeown NE, Harmon KL, Meng XJ, Halbur PG. Porcine circovirus type 2 infection decreases the efficacy of a modified live porcine reproductive and respiratory syndrome virus vaccine. Clin Vaccine Immunol. 2006;13:923–9. http://dx.doi.org/10.1128/CVI.00074-06

2. Lowry R. VassarStats: Website for Statistical Computation [online software]. 2016 Mar 28 [cited 2016 Oct 10]. http://vassarstats.net/

3. Sasseville AM, Boutin M, Gélinas A-M, Dea S. Sequence of the 3′-terminal end (8.1 kb) of the genome of porcine haemagglutinating encephalomyelitis virus: comparison with other haemagglutinating coronaviruses. J Gen Virol. 2002;83:2411–6. http://dx.doi.org/10.1099/0022-1317-83-10-2411

<jrn>4. Carver T, Thomson N, Bleasby A, Berriman M, Parkhill J. DNAPlotter: circular and linear interactive genome visualization. Bioinformatics. 2009;25:119–20. http://dx.doi.org/10.1093/bioinformatics/btn578</jrn>

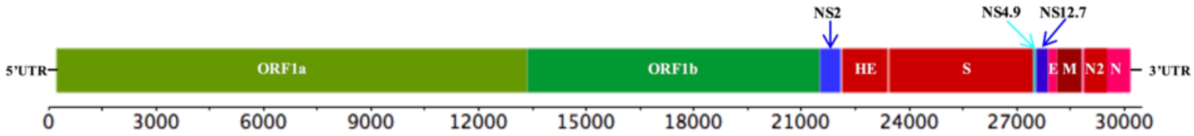

Technical Appendix Figure 1. Schematic representation of the porcine hemagglutinating encephalomyelitis virus (PHEV) genome (PHEV-VW572 strain, GenBank accession no. DQ011855) using DNAPlotter software (*4*). The first two thirds of the PHEV genome are open reading frames (ORF) 1a and 1b encoding the replicase. The remaining genome encodes 6 structural proteins (hemagglutinin-esterase protein, HE; spike glycoprotein, S; envelope protein, E; membrane protein, M; nucleocapsid proteins, N and N2) and three nonstructural proteins (NS2, NS4.9, and NS12.7).

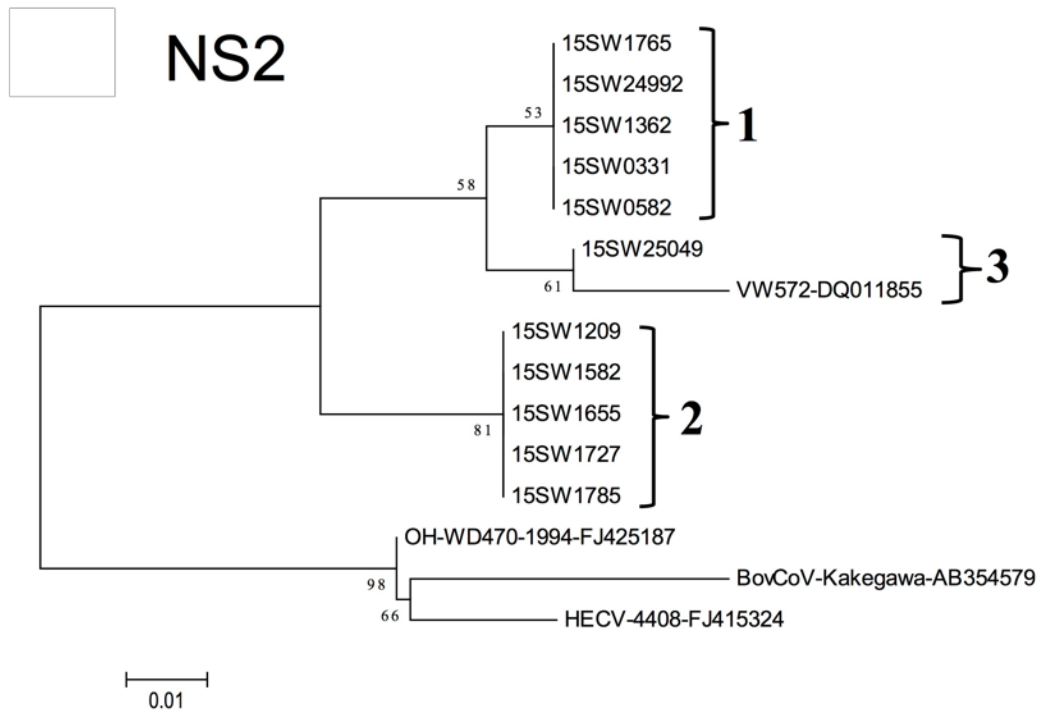

Technical Appendix Figure 2. Phylogenetic tree constructed on the basis of the NS2 gene of porcine hemagglutinating encephalomyelitis virus (PHEV) strains, bovine CoV, human enteric CoV, and white-tail deer CoV. Reference sequences obtained from GenBank are indicated by strain name and accession number. Scale bar represents nucleotide substitutions per site.

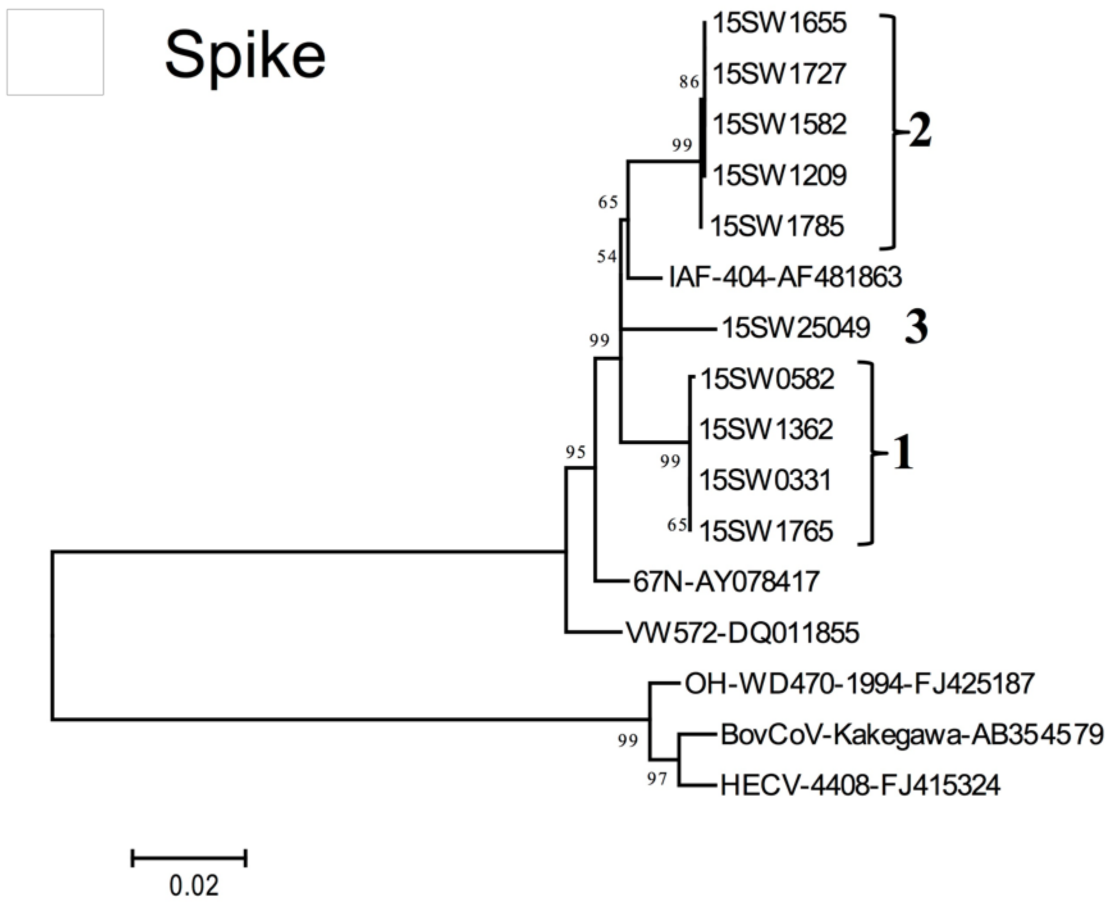

Technical Appendix Figure 3. Phylogenetic tree constructed on the basis of the spike gene of porcine hemagglutinating encephalomyelitis virus (PHEV) strains, bovine CoV, human enteric CoV, and white-tail deer CoV. Reference sequences obtained from GenBank are indicated by strain name and accession number. Scale bar represents nucleotide substitutions per site.

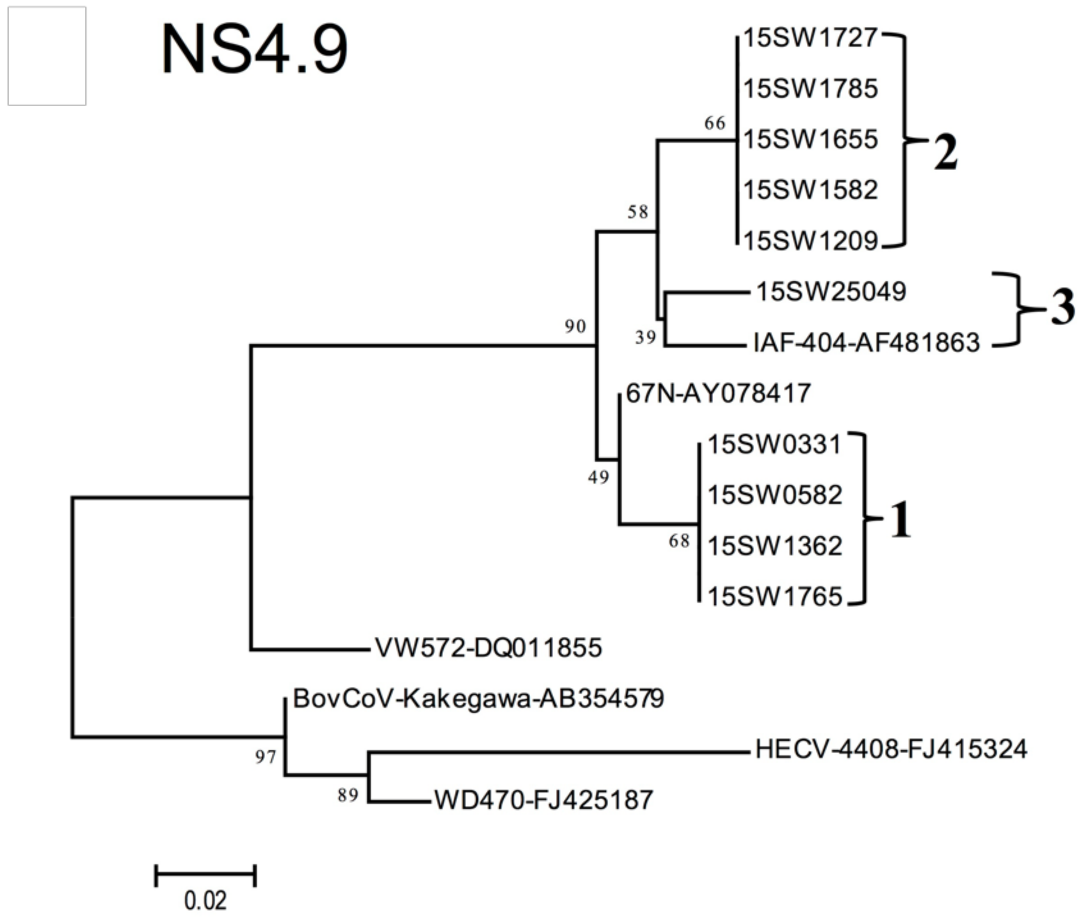

Technical Appendix Figure 4. Phylogenetic tree constructed on the basis of the NS4.9 gene of porcine hemagglutinating encephalomyelitis virus (PHEV) strains, bovine CoV, human enteric CoV, and white-tail deer CoV. Reference sequences obtained from GenBank are indicated by strain name and accession number. Scale bar represents nucleotide substitutions per site.

NS2 sequence

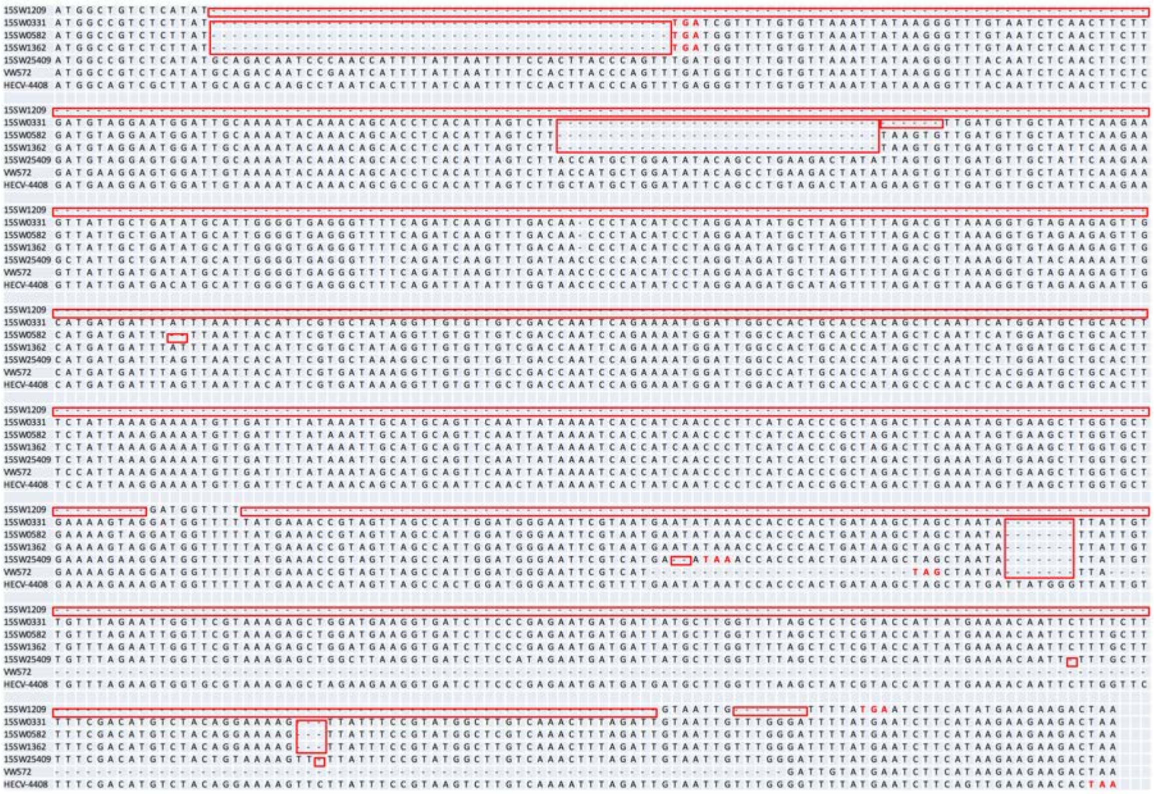

Technical Appendix Figure 5. Nucleotide sequence alignment of NS2 gene of porcine hemagglutinating encephalomyelitis virus (PHEV) strains (15SW1209, 15SW0331, 15SW0582, 15SW1362, 15SW25409, and VW572), and HECV-4408. Deletion regions have been marked with red frames. Stop codons for each strain are in red. MUSCLE alignment program was performed with MEGA.

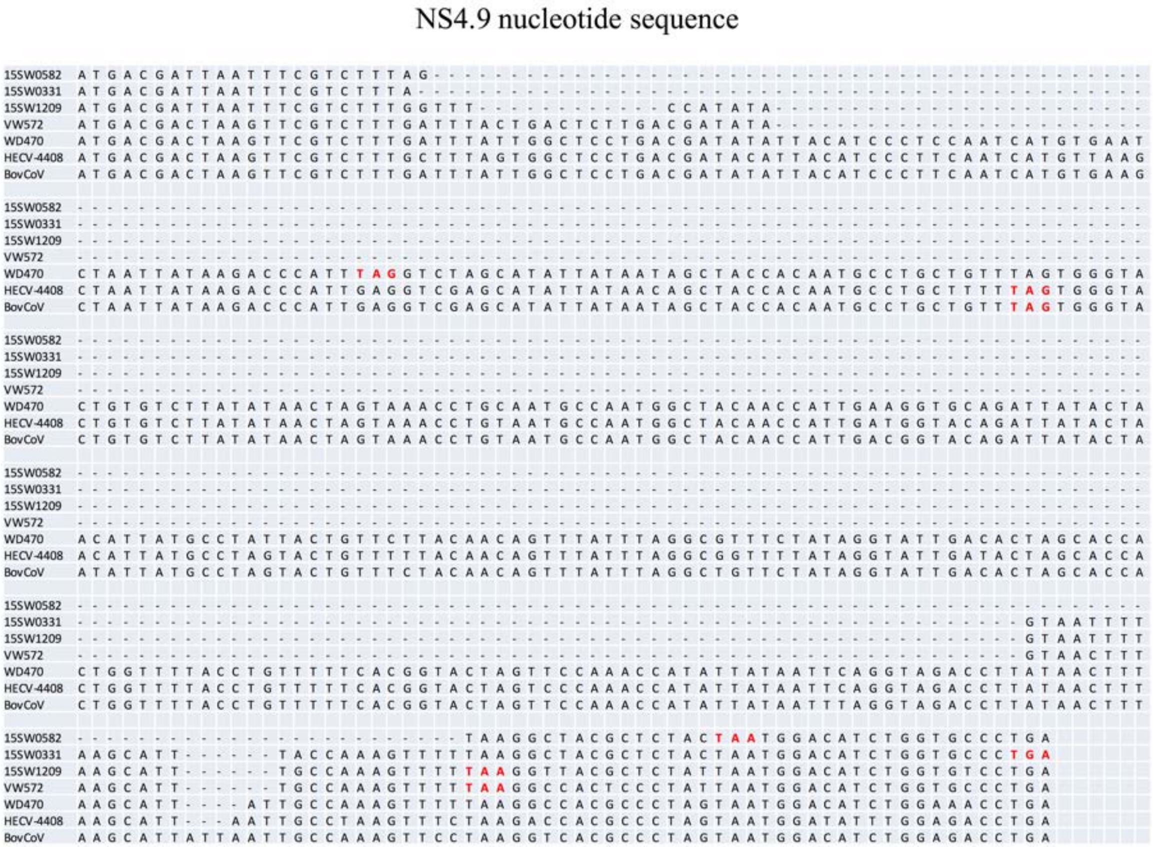

Technical Appendix Figure 6. Nucleotide sequence alignment of NS4.9 gene of porcine hemagglutinating encephalomyelitis virus (PHEV) strains (15SW0582, 15SW0331, 15SW1209, VW572), WD-470, HECV-4408, and BovCoV. Stop codons for each strain are in red. MUSCLE alignment program was performed with MEGA.

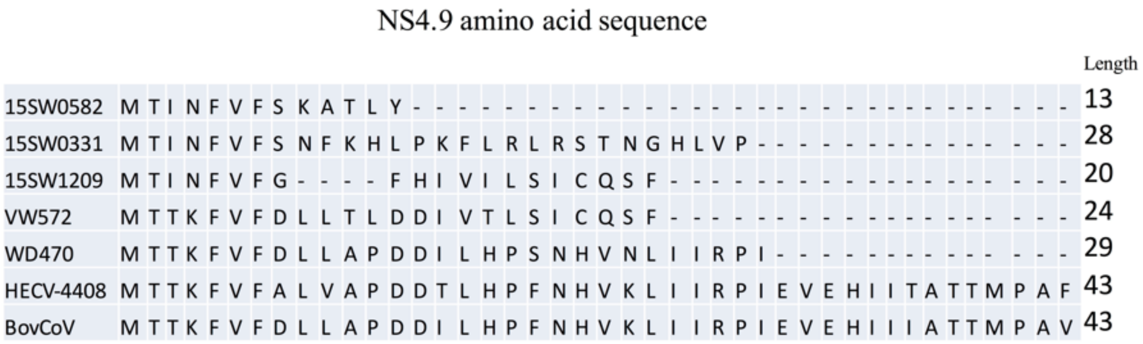

Technical Appendix Figure 7. Amino acid sequence alignment of NS4.9 gene of porcine hemagglutinating encephalomyelitis virus (PHEV) strains (15SW0582, 15SW0331, 15SW1209, VW572), WD-470, HECV-4408, and BovCoV. MUSCLE alignment program was performed with MEGA.
